# Pultruded GFRP Reinforcing Bars Using Nanomodified Vinyl Ester

**DOI:** 10.3390/ma13245710

**Published:** 2020-12-14

**Authors:** Shreya Vemuganti, Rahulreddy Chennareddy, Amr Riad, Mahmoud M. Reda Taha

**Affiliations:** 1 Department of Civil, Construction and Environmental Engineering, University of New Mexico, Albuquerque, NM 87131-0001, USA; svemuganti@unm.edu (S.V.); rahulreddy4@unm.edu (R.C.); 2 Department of Civil Engineering, Faculty of Engineering, Al-Azhar University, Cairo 11371, Egypt; amrriad20@yahoo.com

**Keywords:** pultrusion, GFRP, carbon nanotubes, vinyl ester, shear strength

## Abstract

Glass fiber-reinforced polymer (GFRP) reinforcing bars have relatively low shear strength, which limits their possible use in civil infrastructure applications with high shear demand, such as concrete reinforcing dowels. We suggest that the horizontal shear strength of GFRP bars can be significantly improved by nanomodification of the vinyl ester resin prior to pultrusion. The optimal content of functionalized multiwalled carbon nanotubes (MWCNTs) well dispersed into the vinyl ester resin was determined using viscosity measurements and scanning electron micrographs. Longitudinal tension and short beam shear tests were conducted to determine the horizontal shear strength of the nanomodified GFRP reinforcing bars. While the tensile strength of the GFRP reinforcing bars was improved by 20%, the horizontal shear strength of the bars was improved by 111% compared with the shear strength of neat GFRP bars pultruded using the same settings. Of special interest is the absence of the typical broom failure observed in GFRP when MWCNTs were used. Differential scanning calorimetry measurements and fiber volume fraction confirmed the quality of the new pultruded GFRP bars. Fourier-transform infrared (FTIR) measurements demonstrated the formation of carboxyl stretching in nanomodified GFRP bars, indicating the formation of a new chemical bond. The new pultrusion process using nanomodified vinyl ester enables expanding the use of GFRP reinforcing bars in civil infrastructure applications.

## 1. Introduction

Corrosion is responsible for numerous structurally deficient concrete bridge decks as a result of deicing salts [1]. Glass fiber-reinforced polymer (GFRP) concrete reinforcing bars have become an acceptable alternative for steel bars due to their corrosion resistance [2]. Additionally, GFRP reinforcing bars have remarkable strength and stiffness-to-weight ratio compared with steel and can incorporate fiber optics for sensing [1]. In the last four decades, GFRP reinforcing bars have been widely used for new construction, as well as for strengthening of existing structures. The GFRP pultruded bars have one fiber-dominant (uniaxial) direction, and the two other perpendicular directions are matrix-dominant. The mechanical properties in the matrix-dominant directions are much weaker compared with the fiber-dominant direction [3]. Despite the advantages offered by the corrosion-resistant material, GFRP reinforcing bars have a relatively low interfacial bond between the glass fibers and the polymer matrix [2]. This weak interfacial bond results in several potential limitations of GFRP reinforcing bars, including limited horizontal shear strength, relatively low creep rupture strength, and low fatigue strength [4]. These limitations in mechanical properties result in structural design code provisions limiting the maximum stress allowed in GFRP reinforcing bars [5]. The limited horizontal shear strength of GFRP reinforcing bars restricts their use as dowels for concrete bridge decks and slab on grade and as reinforcement in shear critical regions in reinforced concrete structures [6]. Alternatively, a large number of GFRP reinforcing bars are used, and the design becomes uneconomic. The limited horizontal shear strength of GFRP reinforcing bars, thus, represents a major drawback for widening GFRP field application in civil infrastructure.

The pultrusion process is used for the manufacturing of GFRP reinforcing bars for improved productivity and reducing cost [7,8]. The pultrusion method involves the use of a heated die to integrate fiber reinforcement and resin systems. The resin system essentially behaves like a matrix to bond the fibers together and is important to achieve the durability characteristics and desired mechanical properties offered by the GFRP reinforcing bars [9]. Vinyl ester resin is widely used in the pultrusion of GFRP sections and concrete reinforcing bars in the industry [10,11,12] due to its excellent processability and good mechanical properties [13,14]. Moreover, glass–vinyl ester reinforcing bars have shown high chemical stability when subjected alkaline and acidic environments, attributed to the compact microstructure of the vinyl ester [3,9,15].

Nanomodification of vinyl ester resin systems was reported in the literature to improve its interaction with reinforced fiber, resulting in enhanced fiber reinforced polymer (FRP) composites. Carbon nanotubes (CNTs), one of the strongest materials available today [16], seemed to have significant effects on vinyl ester resins. With appreciable strength and industrial availability, small quantities of multiwalled carbon nanotubes (MWCNTs) were used to improve the strength and stiffness of the polymer composite materials [17]. Surface functionalization of MWCNTs using active chemical groups can be performed to form covalent bonds with the polymer matrix [18]. A detailed discussion about using different dispersion methods and the effect of functionalization of MWCNTs in the epoxy matrix is presented elsewhere [19]. Functionalized MWCNTs were shown to have a chemical reaction with the vinyl ester resin [20]. The nanoscale diameter of MWCNTs allows them to interfere in the polymerization process, altering the final polymer matrix [18].

Laminated FRP composites manufactured using vinyl ester dispersed with carbon nanotubes demonstrated an improved interfacial bond between the resin matrix and the fiber reinforcement [21]. Vinyl ester modified with synthesized CNTs was used in the hand layup fabrication of GFRP composites and showed a 15% improvement in impact strength [22]. Successful nanomodification of vinyl ester resins using MWCNTs was attributed to the existence of styrene as active diluents in vinyl ester [23]. MWCNTs when dispersed in vinyl ester at a ratio beyond percolation limits were shown to form a carbon network that can improve the electrical conductivity of the matrix and, thus, enable in situ damage monitoring [24]. Good dispersion of the nanomaterials in the polymer matrix was reported to control the efficiency of the nanomodification process [23]. Prior work is indicative of the benefits associated with using carbon nanotubes in ester- and epoxy-based FRP laminates manufactured using the vacuum-assisted wet layup technique [25,26]. Furthermore, it was shown that the strength of fiber composites can be improved by using surface-grown CNTs [27] or via surface modification of the fibers along with dispersing CNTs in the sizing polymer [28].

In the current study, pultruded GFRP reinforcing bars using a vinyl ester resin system modified with carboxyl (COOH)-functionalized MWCNTs were produced and tested. No prior work demonstrated the potential use of nanomodified ester-based resins in producing pultruded GFRP concrete reinforcing bars. It is hypothesized that incorporating a small amount of COOH-MWCNTs in the vinyl ester resin system prior to its use in the pultrusion process will enhance the bond between the vinyl ester matrix and the glass fibers, leading to the improved pultruded GFRP reinforcing bars. The suitable amount of MWCNTs for the pultrusion process was first determined based on viscosity measurements and quality of dispersion. The degree of curing was determined using differential scanning calorimetry (DSC), and the fiber volume fraction test was used to evaluate the quality of pultruded GFRP reinforcing bars. Chemical analysis using Fourier-transform infrared spectroscopy (FTIR) was then performed to explain the effect of MWCNTs.

## 2. Materials

For pultrusion, glass fiber spools used were Hybon^®^ 2732 supplied by PPG Industries Ohio, Inc. (Circleville, OH, USA). The glass fiber is categorized as type E with silane as the sizing agent. The glass fiber is compatible with a range of resins such as polyester, vinyl ester, polyurethane, and epoxy resins. The filament diameter is 24 μm and the density of the fiber is 2.58 gm/cc. The vinyl ester resin, with the name 700 Vinyl Ester, including 53.5% vinyl ester and 45% styrene, supplied by US Composites (West Palm Beach, FL, USA) was used in this study. The catalyzation process in the resin was conducted using methyl ethyl ketone peroxide (MEKP, US Composites, West Palm Beach, FL, USA), which essentially serves as a hardening agent. A ratio of 1000 cc of resin to 8 cc of the catalyst (MEKP) was used for the curing process. MWCNTs functionalized with 1.2% carboxylic (COOH) group were used in this study. COOH-MWCNTs with >95 wt.% purity supplied by Cheap Tubes, Inc., Grafton, VT, USA have an inner diameter of 5–10 nm, an outer diameter of 20–30 nm, and length of 10–30 μm with a bulk density of 0.28 gm/cc and specific surface area of 110 m^2^/gm.

## 3. Pre-Pultrusion Preparation and Analysis Methods

To evaluate the suitability of the vinyl ester resin for the pultrusion process when modified with COOH functionalized MWCNTs, viscosity tests of neat vinyl ester resin and MWCNTs-dispersed vinyl ester nanocomposite were conducted. This is because the viscosity of the resin is one of the most critical parameters in the pultrusion process [29]. Four contents of MWCNTs were examined: 0.0% (neat), 0.5 wt.%, 1.0 wt.%, and 2.0 wt.%. All MWCNTs mixing ratios were considered as a weight percentage of the vinyl ester resin. To gain insight into the dispersion of the MWCNTs–vinyl ester nanocomposite, scanning electron microscope (SEM) investigations were also conducted.

The dispersion of COOH-MWCNTs in the vinyl ester resin was performed as a two-stage process involving mechanical stirring and ultrasonication, which are considered common techniques in polymer processing [30]. In the first stage, each content of MWCNTs was added to the vinyl ester resin. Mechanical stirring at 80 °C for 120 min at 800 rpm was performed. Stage 1 was followed by Stage 2 in which ultrasonication at 40 °C for 60 min was performed after 5 min of degassing to disperse the MWCNTs in the vinyl ester resin [31,32]. Following the dispersion process, the MWCNTs–vinyl ester nanocomposite was cooled down to room temperature prior to any testing.

In the pultrusion process, achieving fiber wet-out gets challenging as the resin viscosity increases because a non-viscous resin offers less resistance to flow compared with a viscous resin [33]. When MWCNTs are introduced in a polymer resin, the flow characteristics of the resin are significantly altered due to their high aspect ratio [34]. Rheological evaluation of the MWCNT–vinyl ester nanocomposite to evaluate the resin flow behavior was performed using an RST-Coaxial Cylinder Rheometer (Ametek Brookfield, Middleboro, MA, USA). An RST CCT-40 spindle (Ametek Brookfield, Middleboro, MA, USA) was used with a viscosity range of 0.0003–27.6 K·Pa·s and a shear rate of 0.0215–2.79 K·s^−1^. A controlled rate operation at 1–500 s^−1^ was performed on a sample volume of 68.5 mL of the MWCNTs–vinyl ester nanocomposite. Resin shear stress values were obtained and plotted against shear rate to achieve a linear behavior, the slope of which indicates the viscosity of the MWCNT–vinyl ester polymer nanocomposite.

During the dispersion of MWCNTs in the vinyl ester resin, the evaporation of styrene results in producing variable concentrations of the nanomaterials across the resin [24]. As a result, poor dispersion due to MWCNTs entanglement is possible. Such poor dispersion can lead to poor mechanical behavior. To evaluate the dispersion of different MWCNTs contents in the vinyl ester resin, SEM investigations of hardened MWCNTs–vinyl ester nanocomposite incorporating different weight ratios of MWCNTs were performed. The uniformly mixed polymer was poured into 5 mm thick, 5 mm wide, and 25 mm long silicone molds and cured at 80 °C for 12 h. The specimens were demolded from the silicone molds, and SEM investigations were conducted using a VEGA3 thermionic emission SEM system by TESCAN (Brno, Czech Republic). To enhance conductivity, the specimens were polished and sputter-coated with gold/palladium (Au/Pd). MWCNTs spotted in the micrographs provided insight into the dispersion levels of the vinyl ester nanocomposite.

## 4. Pultrusion Process

**Figure 1:**
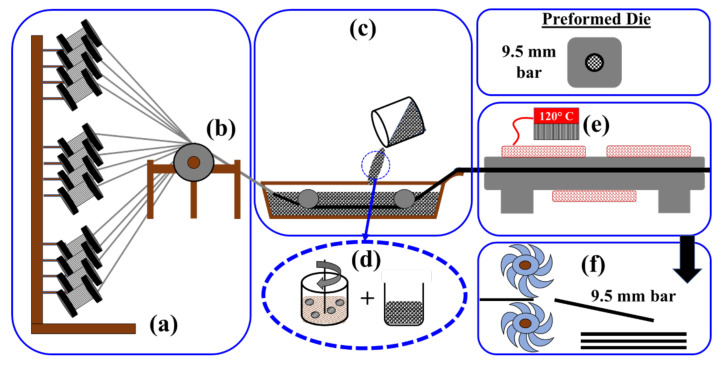
Schematic describing the pultrusion process: (**a**) fiberglass roving provision; (**b**) preforming; (**c**) open bath impregnation with nanomodified resin; (**d**) nanomodified resin synthesis; (**e**) curing die with heating plates; (**f**) take-off unit.

The pultrusion process used in this study incorporates four principal steps, which are (1) provision of fiberglass roving and preforming, (2) open bath impregnation, (3) curing process in die, and (4) take-off unit [29]. The pultrusion process was performed using a speed-controlled gear motor running at a constant pull speed of 3.0 mm/s. This particular pull speed was chosen to ensure adequate heat supply for proper curing, following recommendations by previous studies [35,36]. However, it is essential to note that, if a commercial product is to adopt the method proposed herein, a relatively high pull speed of at least 8.0 mm/s might be necessary. The pultrusion process incorporating the nanomodification of the vinyl ester resin is shown schematically in Figure 1.

**Figure 2:**
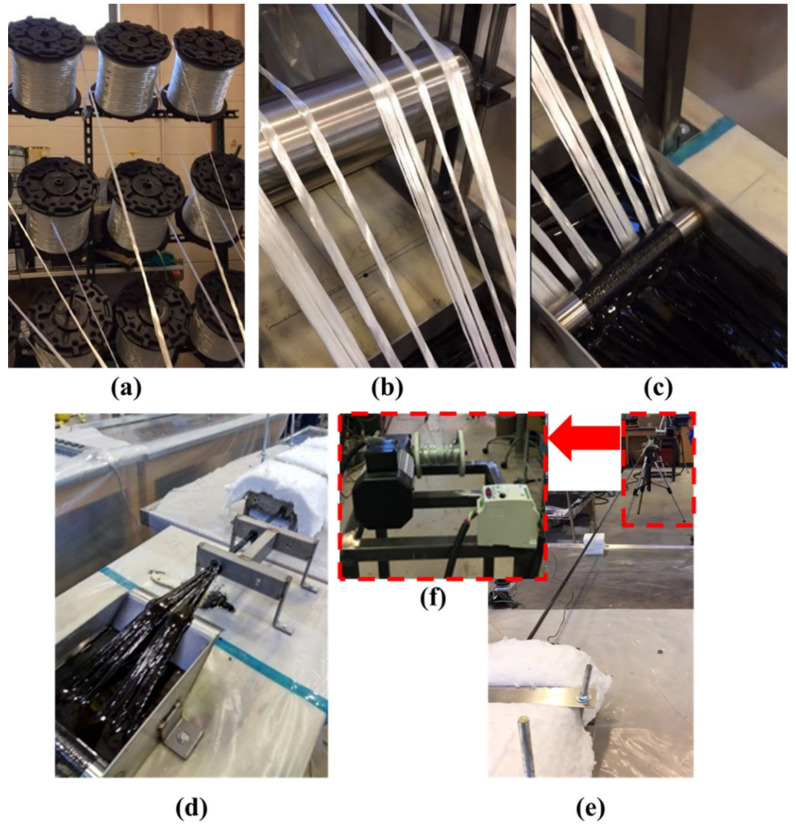
(**a**) Provision of fiberglass roving; (**b**) preforming; (**c**) open bath impregnation; (**d**) guiding and curing process in die; (**e**) take-off unit; (**f**) motor.

Twelve fiberglass rovings were used in the pultrusion process. The fiberglass was collected onto a horizontal steel roller fixed to a steel stand with welded supports. From the roller, the fiberglass was pulled into the impregnation bath under fixed horizontal rollers to ensure the tension of the strands and complete saturation and wet-out of the fibers. The saturated fiberglass was pulled into two steel guides placed at a 150 mm distance with holes of 9.5 mm diameter. For the curing process, the fiberglass was pulled into a 600 mm long steel die with a 9.5 mm diameter hole. The die was provided with heating plates and coils connected to a temperature regulator to maintain a temperature of approximately 120 °C inside the die to facilitate the curing process. The cured bar was pulled out with gripping devices and cut with a saw. The five specimens tested were randomly selected from numerous independent rounds of pultrusion to ensure reproducibility of the pultruded GFRP bars. The specimens were stored in the laboratory with a controlled temperature maintained at 23 °C ± 1 °C and a relative humidity of 50% ± 2%. Figure 2 illustrates the pultrusion process as performed in our laboratory facility.

## 5. Post-Pultrusion Experimental Testing and Analysis Methods

### 5.1. Degree of Cure

The degree of cure of MWCNTs–vinyl ester nanocomposite for sufficient curing of the pultruded GFRP bars was evaluated as per the ASTM E2160–04 Standard test method for heat of reaction of thermally reactive materials by differential scanning calorimetry (DSC) [37]. For a thermoset resin, the degree of cure is determined using Equation (1).

(1) $$\mathrm{Degree}\;\mathrm{of}\;\mathrm{Cure}\;=\;\frac{H_{Uncured}-H_{cured}}{H_{uncured}}\times 100\%$$

where $H_{Uncured}$ represents the weight-normalized heat of reaction of uncured resin, and $H_{cured}$ represents the weight-normalized heat of reaction of the cured specimen for which the degree of cure is being calculated. Specimens for DSC measurements were fabricated using the neat and dispersed mix of MWCNT–vinyl ester nanocomposite mixed with the MEKP hardening agent. The uniformly mixed resin was poured into 5 mm thick, 5 mm wide, and 25 mm long silicone molds and cured at 80 °C for 12 h. These cured specimens were demolded from the silicone molds, and 2 mg of sample was weighed to within ±1 μg in a Tzero aluminum pan (TA Instruments, New Castle, DE, USA). An uncured vinyl ester specimen was obtained by mixing a neat vinyl ester resin with MEKP. The specimens were heated at 10 °C/min from ambient temperature to 400 °C under a nitrogen environment using a DSC 250 by T.A. Instruments (New Castle, DE, USA). The integration of the exothermic heat flow curve recorded as a function of temperature was obtained, indicating the heat of reaction.

### 5.2. Fiber Volume Fraction and Microcopy

The fiber volume fraction of the pultruded GFRP bars with and without MWCNTs was determined using the ASTM D3171-15 Standard test methods for constituent content of composite materials [38] by the hot furnace combustion method. The fiber content, as the volume fraction percentage, was calculated using Equation (2). A Celestron Handheld Digital Microscope (Celestron, Torrance, CA, USA) with 5.0 MP camera at 20× power was used to inspect the cross-section of the pultruded bars to observe any voids.

(2) $$\mathrm{Volume}\;\mathrm{fraction}\;V_{f}=\left(\frac{M_{f}}{M_{i}}\right)\times 100\%\times \frac{\rho_{c}}{\rho_{r}}$$

where $M_{i}$ is the initial mass (gm) of the specimen, $M_{f}$ is the final mass (gm) of the specimen after combustion, $\rho_{r}$ is the density (gm/cc) of the reinforcement, and $\rho_{c}$ is the density (gm/cc) of the specimen.

### 5.3. Fourier-Transform Infrared Spectroscopy (FTIR)

To investigate the potential of a chemical reaction between COOH-MWCNTs and the vinyl ester resin, FTIR measurements were performed. Specimens for FTIR measurements were fabricated using the neat and dispersed mix of MWCNT–vinyl ester nanocomposite hardened with the MEKP hardening agent. The uniformly mixed resin was poured into 5 mm thick, 5 mm wide, and 25 mm long silicone molds and cured at 80 °C for 12 h and then demolded. A NICOLET 6700 (Thermo Fisher Scientific Inc., Waltham, MA, USA) with an attenuated total reflectance (ATR) cell attachment was used to perform the FTIR measurements. Specimens were powdered to suit the ATR cell attachment. The Omnic^TM^ software package (Thermo Scientific OMNIC^TM^ series software, 8. 3. 103, Thermo Fisher Scientific Inc., Waltham, MA, USA) was used for data collection, peak fitting, and analysis. Spectra were background-corrected, and a total of 64 scans were performed on each sample to obtain the transmittance from 4000–400 cm^−1^ wave number range at a resolution of 1.928 cm^−1^ wave number. The transmittance (*T*) was determined from the ATR spectroscopy using the Beer–Lambert Law as presented in Equation (3), where I and $I_{0}$ are the intensities of the transmitted and incident radiation, respectively [39]. FTIR analysis was conducted on the spectra to identify the peaks formed by the characteristic chemical reaction between COOH-MWCNTs and the vinyl ester resin.

(3) $$T=\left(\frac{I}{I_{0}}\right)$$

### 5.4. Mechanical Testing of the Pultruded GFRP Bars

**Figure 3:**
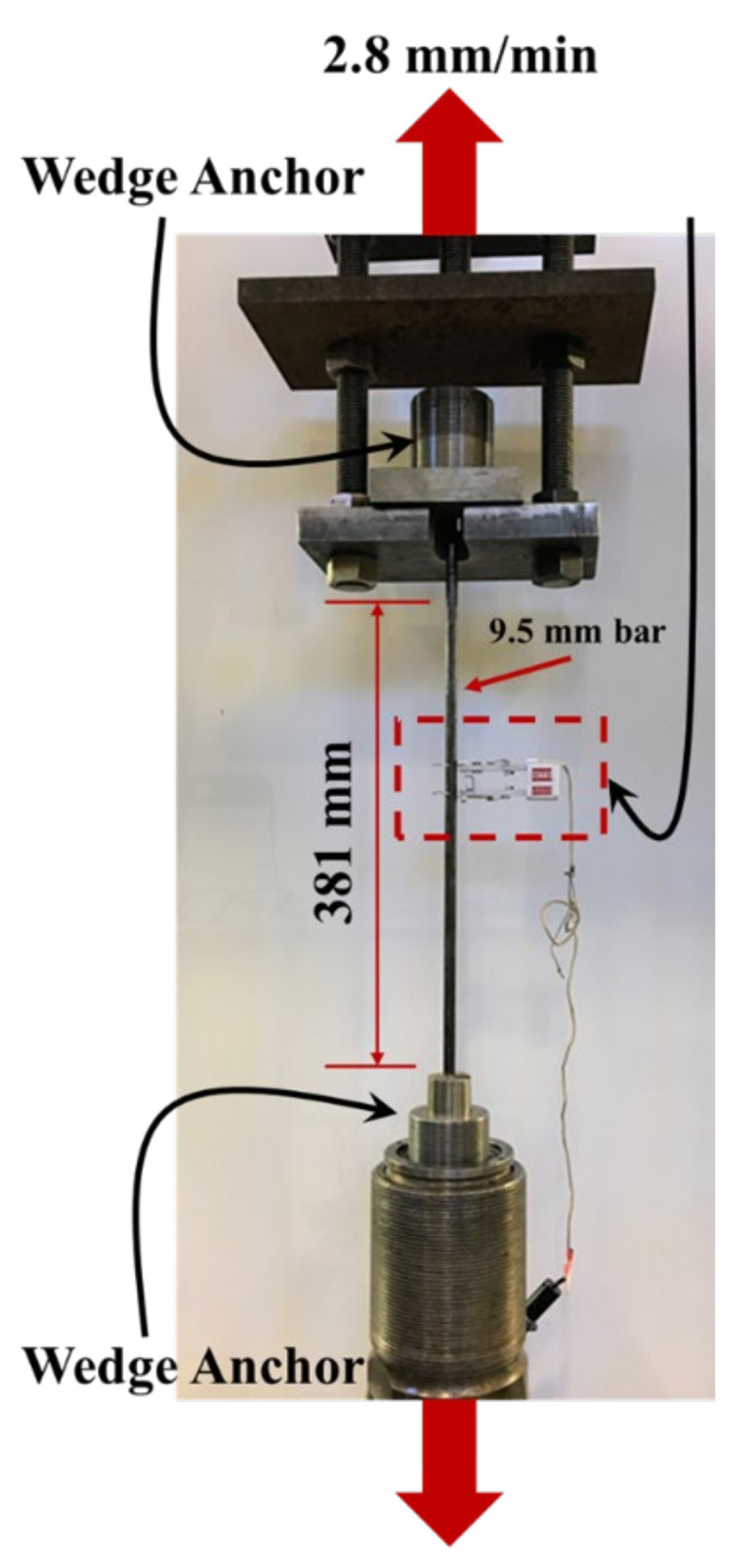
Longitudinal tension test setup of pultruded GFRP bars using mechanical wedge anchors.

Longitudinal tension tests were performed on the pultruded GFRP bars using an Instron^®^ loading frame (Instron Corporation, Norwood, MA, USA) with 530 kN capacity. Three bars of pultruded neat GFRP and three bars of pultruded GFRP bars incorporating 0.5 wt.% COOH-MWCNTs were tested under longitudinal tension test at a crosshead displacement rate of 2.8 mm/min following the ASTM D7205/D7205M Standard test method for tensile properties of fiber reinforced polymer matrix composite bars [40]. The displacement-controlled protocol was chosen to produce an approximate loading rate between 100 and 500 MPa/min. All the specimens failed within 10 min of loading. To prevent stress concentrations at the bar ends, stainless-steel wedge anchors were used as per ASTM D7205/D7205M. The anchoring system used in this study is based on patented stainless-steel anchors shown to be successful under static and fatigue loading [41]. The diameter of the bars was 9.5 mm (denoted as imperial bar size #3), and the length of the bars between the anchors was 381 mm. An MTS contact extensometer with a 25.4 mm gauge length was attached to the bar to serve as a strain indicating device. The tensile strength and modulus of elasticity were calculated using Equations (4) and (5).

(4) $$\sigma_{t}=\frac{P_{max}}{A}$$

(5) $$E=\frac{∆\sigma }{∆\varepsilon }$$

where $\sigma_{t}$ is the tensile strength, $P_{max}$ is the maximum force prior to failure, A is the cross-sectional area of the bar, E is the tensile modulus of elasticity, ∆σ is the difference in applied tensile stress between the starting and ending strain points, and ∆ε is the difference in the average tensile strain between the starting and ending strain points. The start and end strain values are the 25% and 50% strains of the ultimate failure strain, respectively, and their corresponding stress values based on ASTM D7205 [40]. The longitudinal tension test setup is shown in Figure 3.

Several tests have been reported in the literature to determine the interlaminar shear strength of FRP laminates [42,43]. However, the short beam shear test based on the ASTM D4475 is a relatively easy and inexpensive test [11,42,44]. In the short beam shear test, the span-to-depth ratio is kept between 3 and 8 to ensure interlaminar shear failure [44,45]. In this paper, short beam shear tests were performed on the pultruded GFRP bars using an MTS^®^ Bionex servo-hydraulic system (MTS Systems Corporation, Eden Prairie, MN, USA) with 25 kN capacity. Five bars of pultruded neat GFRP and five bars of pultruded GFRP bars incorporating 0.5 wt.% COOH-MWCNTs were tested under horizontal shear at a crosshead displacement rate of 1.3 mm/min following ASTM D4475-16 Standards for apparent horizontal shear strength of pultruded reinforced plastic rods using the short beam method [44]. The specimens for the short beam test were 50 mm long obtained by cutting the pultruded bars. The shear specimen span-to-diameter ratio was maintained as 3:1 to achieve a length of 28.6 mm between the supports such that the loading was applied at the middle of the specimen. The short beam shear strength was calculated using Equation (6).

(6) $$S=\frac{0.849\times P}{d_{b}^{2}}$$

where S is the apparent horizontal shear strength, P is the breaking load, and $d_{b}$ is the bar diameter.

## 6. Results and Discussion

### 6.1. Results from Rheological Evaluations

**Figure 4:**
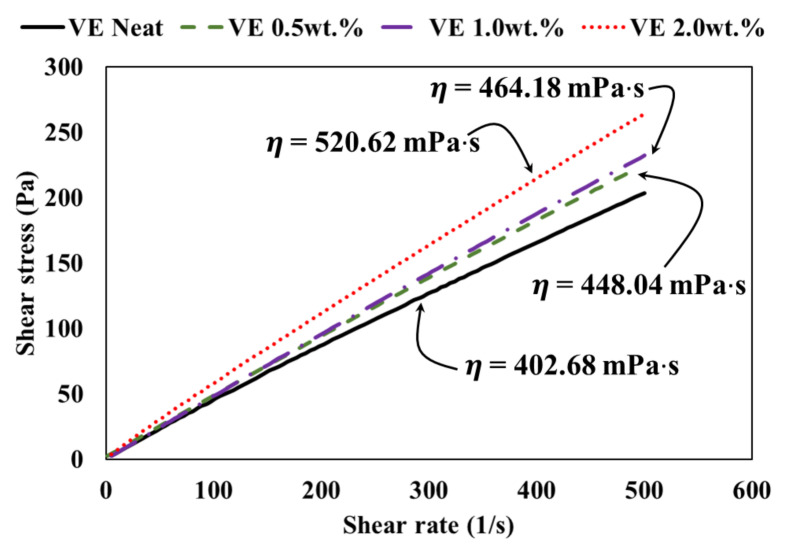
Vinyl ester resin shear stress versus shear rate with varying MWCNTs contents.

The resin shear stress plotted against shear rate for each vinyl ester type with 0.0 wt.%, 0.5 wt.%, 1.0 wt.%, and 2.0 wt.% COOH-MWCNTs is shown in Figure 4. The slope of the line represents the viscosity, as indicated in Figure 4.

Compared with the neat vinyl ester with 0.0 wt.% COOH-MWCNTs, the viscosity of vinyl ester incorporating 0.5 wt.%, 1.0 wt.%, and 2.0 wt.% COOH-MWCNTs increased by 11%, 15%, and 30%, respectively. The increase in viscosity due to the addition of COOH-MWCNTs is attributed to particle–particle interactions of the MWCNTs, leading to increased particle friction with the resin [20]. The increase in viscosity of vinyl ester incorporating 0.5 wt.% and 1.0 wt.% COOH-MWCNTs was relatively small, while the increase in viscosity of vinyl ester incorporating 2.0 wt.% COOH-MWCNTs was relatively high. The limited increase in viscosity did not affect the resin suitability for the pultrusion process. However, the 30% increase in vinyl ester resin incorporating 2.0 wt.% MWCNTs had a negative effect on the pultrusion process and would not allow efficient fiber wet-out. All viscosity measurements of the vinyl ester with 0.0 wt.%, 0.5 wt.%, 1.0 wt.%, and 2.0 wt.% COOH-MWCNTs are presented in Table 1.

### 6.2. Results from SEM Investigations

**Figure 5:**
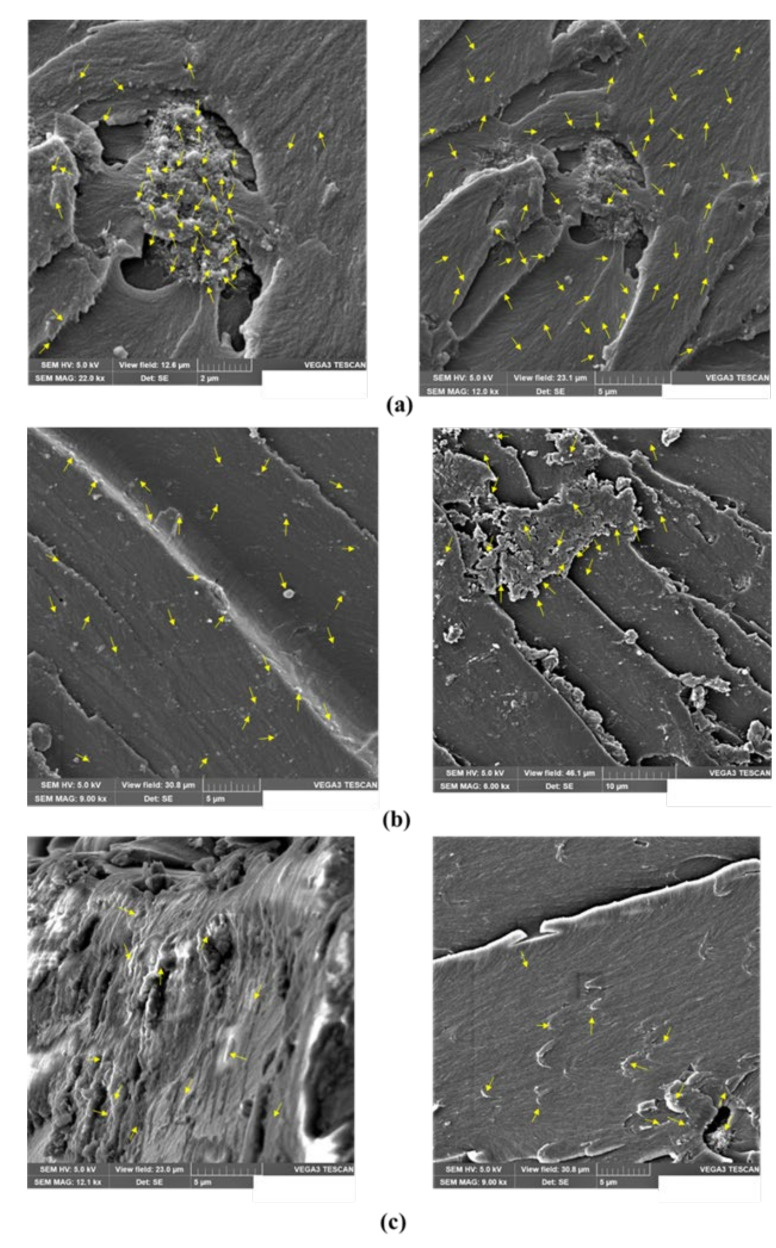
Scanning electron micrograph showing hardened vinyl ester incorporating (**a**) 0.50 wt.%, (**b**) 1.0 wt.%, and (**c**) 2.0 wt.% COOH-MWCNTs with (**left**) high magnification (**right**) low magnification.

To gain insight into the dispersion of the vinyl ester blend with different contents of MWCNTs suspensions, SEM micrographs of COOH-MWCNTs–vinyl ester nanocomposites with 0.5 wt.%, 1.0 wt.%, and 2.0 wt.% MWCNTs are shown in Figure 5. SEM micrographs were acquired with an accelerating voltage of 5.0 kV. This voltage is suitable for the visualization of CNTs as bright features in a polymer matrix [46]. A uniform distribution of MWCNTs was only observed in vinyl ester incorporating 0.5 wt.% COOH-MWCNTs. The MWCNTs in the vinyl ester polymer matrix are represented by arrows, as shown in Figure 5a. This supports the earlier observation of a limited increase in initial viscosity of vinyl ester incorporating 0.5 wt.% COOH-MWCNTs compared with all other COOH-MWCNTs contents. Figure 5b shows bright features representing clusters of MWCNTs in the vinyl ester matrix, which can be explained by the relatively poor dispersion of this high content of MWCNTs due to increased viscosity. Furthermore, Figure 5c also shows the clusters and the potential of MWCNTs entanglement forming agglomerates due to the relatively higher content of 2.0 wt.% MWCNTs in the vinyl ester matrix. Carbon nanotubes entanglement can occur at high concentrations of MWCNTs due to the evaporation of a styrene monomer in vinyl ester during the dispersion process. It is evident from the microstructural analysis that high concentrations of MWCNTs, including 1.0 wt.% and 2.0 wt.%, are not suitable for use in the pultrusion process due to an increase in viscosity and absence of good dispersion. Therefore, vinyl ester resins incorporating 1.0 wt.% and 2.0 wt.% MWCNTs were excluded from the further investigation as these mixtures were deemed unsuitable to produce pultruded GFRP bars. Neat vinyl ester and vinyl ester nanocomposite incorporating 0.5 wt.% COOH-MWCNTs were used to pultrude GFRP bars. Finally, while not used herein, we note that plasma etching might be an effective technique in producing high-quality micrographs of SEM specimens incorporating MWCNTs [47].

### 6.3. Results of Fiber Volume Fraction, Microscopy, and Cure Characteristics

**Figure 6:**
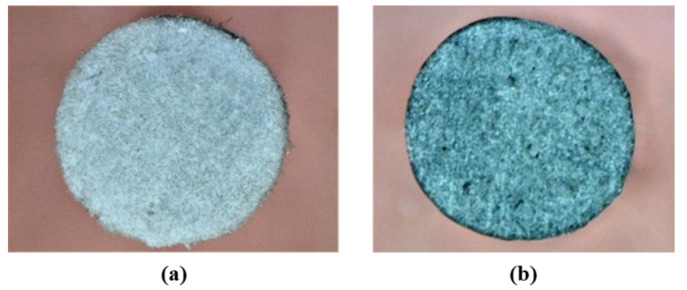
Microscopic images at 20× magnification showing cross-section of (**a**) neat pultruded GFRP bar and (**b**) pultruded GFRP bar incorporating 0.5 wt.% COOH-MWCNTs.

The fiber volume fractions of the pultruded neat GFRP and pultruded GFRP bars incorporating 0.5 wt.% COOH-MWCNTs were 61.2% and 59.3%, respectively. The two bars include acceptable and similar fiber volume fractions. Microscopic images of the cross-sections of a neat pultruded bar and a pultruded bar with 0.5 wt.% COOH-MWCNTs are shown in Figure 6. The figure shows the absence of large voids and the similarity of the cross-section of both bars.

**Figure 7:**
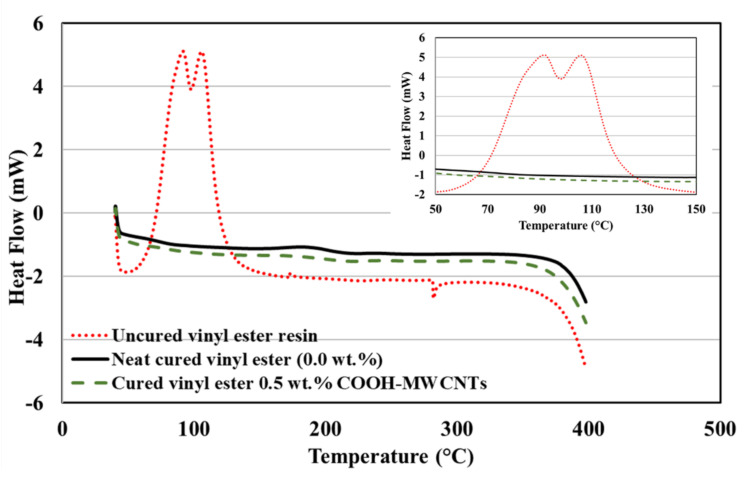
Differential scanning calorimetry graphs for uncured neat vinyl ester with MEKP, neat cured vinyl ester, and cured vinyl ester incorporating 0.5 wt.% COOH-MWCNTs. The inset figure shows a close look at the behavior of the three materials at the peak heat flow.

The DSC heat flow curves of the uncured neat vinyl ester resin and cured vinyl ester with 0.0 wt.% and 0.5 wt.% COOH–MWCNTs are shown in Figure 7, and the heat of reaction values are listed in Table 2. The uncured neat vinyl ester showed two major peaks in the range of 50–150 °C with an associated enthalpy of normalized heat of reaction about 578 J/g, as reported in the literature [48]. The two peaks observed in the DSC heat flow curves can be explained by the change in the degree of curing of the uncured vinyl ester during the DSC measurements. As the temperature increased, the degree of curing first increased, reaching the first peak at ~90 °C, and then decreased. The increasing temperature reduced the uncured resin viscosity, providing the space for the free radicals to move and to start polymerization of the resin. Consequently, the polymerization progressed further and led to another increase in curing, resulting in the second peak at ~110 °C. Similar observations were reported by Zhang et al. [49].

A significant decrease in enthalpy values was observed for both neat and vinyl ester incorporating 0.5 wt.% COOH-MWCNTs, which was attributed to the elimination of residual reactions, resulting in a complete reaction because of the curing process. The enthalpy of 13.1 J/g for neat vinyl ester and enthalpy of 10.9 J/g for vinyl ester with 0.5 wt.% COOH-MWCNTs are comparatively similar and much lower than the uncured specimens indicating complete reaction and a high degree of cure, as presented in Table 2. While an accurate determination of the glass-transition temperature (T_g_) using DSC has proven challenging compared to other methods (e.g., dynamic mechanical analysis) [50], incorporating MWCNTs in vinyl ester has been reported to result in increasing T_g_ by a few degrees [21,51]. Such an increase in T_g_, while limited, is beneficial for improving the fire resistance of GFRP reinforcing bars when used in civil infrastructure.

### 6.4. Results of FTIR Spectral Analyses

**Figure 8:**
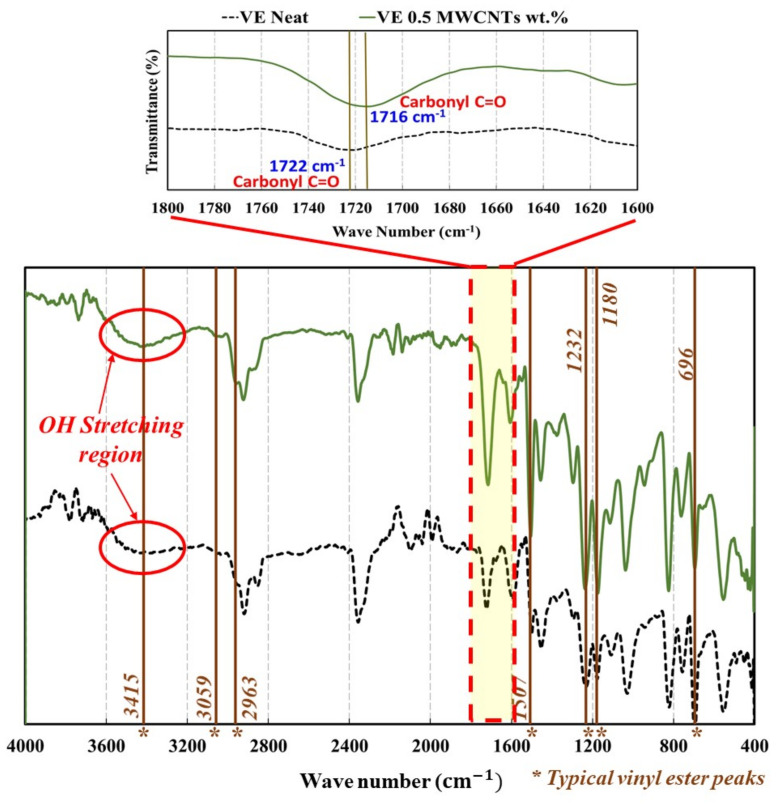
Fourier-transform infrared (FTIR) spectrogram showing bands observed for neat vinyl ester and vinyl ester incorporating 0.5 wt.% COOH-MWCNTs.

In the FTIR spectroscopy shown in Figure 8, a chemical reaction in FTIR spectral analyses can be identified by observing the shift in vibration peaks. Typical bands of vinyl ester were found at 3415 ${\mathrm{cm}}^{-1}$ for the OH hydroxyl group, 3059 ${\mathrm{cm}}^{-1}$ for the CH aromatic benzene ring, 2963 ${\mathrm{cm}}^{-1}$ for the CH aliphatic, 1507 cm^−1^ for the CH aromatic benzene ring, 1232 cm^−1^ for the C–O ester, 1180 cm^−1^ for the C=O ether, and 696 cm^−1^ for the CH monosubstituted aromatic benzene ring [52]. In COOH-MWCNTs, there are normally three distinctive bands, which are C=O, O–H, and C–O [53]. For carboxylic acid (COOH), the typical band is observed at 1700 cm^−1^. In COOH-MWCNTs, this band is observed at around 1710–1735 cm^−1^ [54]. Bands at 1722 cm^−1^ and 1621 cm^−1^ were attributed to the bond stretching of carbonyl group C=O and carboxylic C–O groups [55]. The C=O peak was also identified at 1719 cm^−1^ [56]. C=O stretching has also been reported at 1725 cm^−1^ [55]. The C–O peak was also observed strongly around 1644 cm^−1^ [53]. This peak may also appear in some cases at 1565 cm^−1^ indicating the carboxylate anion stretch mode [57]. When COOH-MWCNTs are added to resins, the C=O stretch has been reported around 1735 cm^−1^ with a peak at 1734.4 cm^−1^ [58,59].

In Figure 8, the FTIR spectra of neat vinyl ester and vinyl ester incorporating 0.5 wt.% COOH-MWCNTs are presented, and the important bands are highlighted. The FTIR analysis of both neat and vinyl ester incorporating 0.5 wt.% COOH-MWCNTs showed typical peaks at 3415 ${\mathrm{cm}}^{-1}$, 3059 cm^−1^, 2963 cm^−1^, 1507 cm^−1^, 1232 cm^−1^, 1180 cm^−1^, and 696 cm^−1^ as expected. The O–H stretching region was observed in both neat and 0.5 wt.% COOH-MWCNTs–vinyl ester nanocomposite in the range of 3300 to 3550 cm^−1^. Similar O–H absorption peaks appeared at 3415 cm^−1^ for both materials. This observation indicates that most of the hydroxyl groups of the COOH-MWCNTs were consumed via esterification reaction with the secondary hydroxyl groups of the vinyl ester [60]. Furthermore, the stretch position of carbonyl C=O of the neat vinyl ester at 1722 cm^−1^ was shifted to 1716 cm^−1^ in the presence of COOH-MWCNTs and its feature appears greatly enhanced. This can be noted by comparing the absorption ratios of the C–O stretch at 1594 cm^−1^ to the carbonyl C=O stretches at 1716 cm^−1^ and 1722 cm^−1^. The increased carbonyl peak intensity in the presence COOH-MWCNTs versus the neat resin refers to a chemical interaction between the hydroxyl group of the vinyl ester and COOH-MWCNTs [61]. These differences observed in the FTIR spectrogram peak locations were attributed to the occurrence of chemical reactions due to the interaction between COOH-MWCNTs and the vinyl ester matrix.

### 6.5. Results from Mechanical Testing

**Figure 9:**
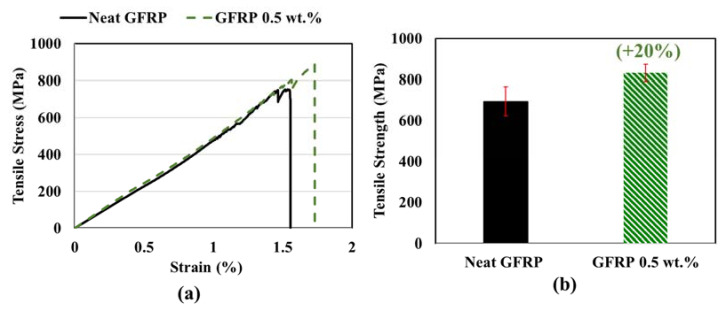
Longitudinal tension test: (**a**) representative stress–strain behavior; (**b**) comparison of the tensile strength of pultruded GFRP bars made with neat vinyl ester and 0.5 wt.% COOH-MWCNTs–vinyl ester nanocomposite. The percentage above the error bar shows the per percentage improvement compared with neat GFRP.

The stress–strain behavior of pultruded GFRP bars produced using vinyl ester incorporating 0.0 wt.% and 0.5 wt.% of COOH MWCNTs is shown in Figure 9. The stress–strain behavior of GFRP incorporating MWCNTs showed a linear elastic behavior to failure. All the GFRP specimens showed similar slopes with and without MWCNTs was observed. Compared with the neat pultruded GFRP bars, a nonsignificant increase in the elastic modulus was observed in pultruded GFRP bars with 0.5 wt.% COOH MWCNTs vinyl ester nanocomposite. The strain at failure was higher for the specimens made with 0.5 wt.% COOH-MWCNTs, as shown in Figure 9. The manufacturer’s reported elastic modulus of the E-glass fibers is 72.4 GPa. For the fiber volume fraction of 61.2% as measured herein for the neat GFRP pultruded bars, and with the observed failure strain of the neat GFRP pultruded bars of 0.0155 mm/mm, the tensile strength can be predicted using basic principles of composite mechanics to be 686.7 MPa [62]. The experimentally observed mean tensile strength of the neat GFRP pultruded bars is 694 MPa, which is very close to the predicted tensile strength.

**Figure 10:**
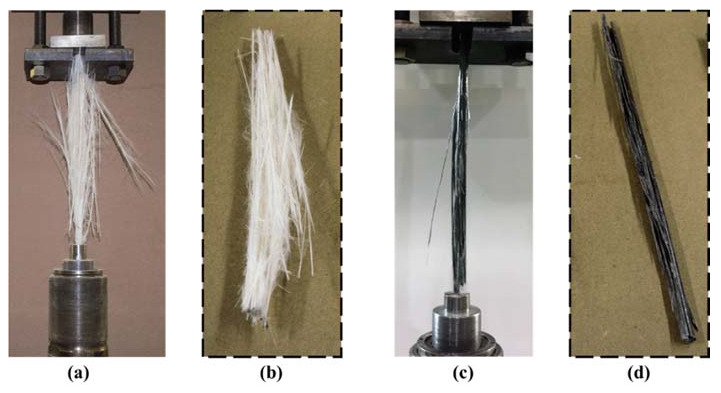
Longitudinal tension failure modes of pultruded GFRP bars with (**a**) complete setup of neat pultruded GFRP bars, (**b**) failed specimen of neat pultruded GFRP bars, (**c**) complete setup of pultruded bars with 0.5 wt.% COOH-MWCNTs and (**d**) failed specimen of pultruded bars with 0.5 wt.% COOH-MWCNTs

Tension test results indicated that an improvement of 20% in tensile strength in pultruded GFRP bars with 0.5 wt.% COOH MWCNTs when compared with neat pultruded GFRP. This improvement was proven to be statically significant, with a 95% confidence level using the Student *t*-test. More interestingly, the different modes of tension failure observed of pultruded neat GFRP bars and pultruded GFRP bars incorporating 0.5 wt.% COOH-MWCNTs are shown in Figure 10. It is apparent that neat GFRP showed typical broom failure, as reported in the literature [2]. However, pultruded GFRP incorporating 0.5 wt.% COOH-MWCNTs showed almost no broom failure. We suggest that this change in tensile strength and the tension failure mode is related to the ability of MWCNTs to have mechanical and chemical effects. First, MWCNTs work as microfibers resisting crack propagation and improve the interlaminar fracture toughness of the GFRP composite [25]. Second, the functionalization of the MWCNTs with the carboxylic COOH group allows them to react with the silane sizing on the surface and, thus, improve the chemical bond. The combined mechanical and chemical effects result in increasing the tensile strength and limiting the broom effect that follows fibers debonding from the matrix.

**Figure 11:**
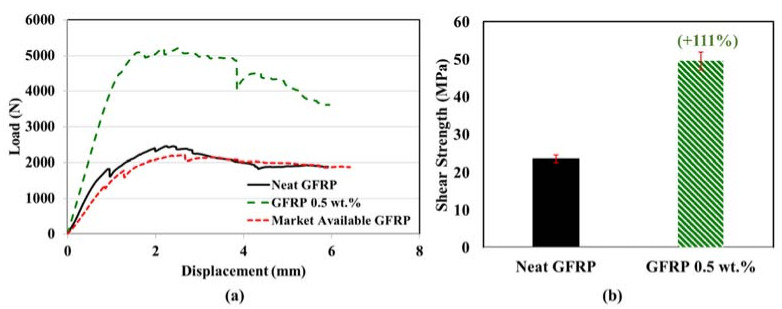
Short beam shear test for horizontal shear strength of pultruded GFRP: (**a**) representative load–displacement behavior of the neat GFRP, GFRP including 0.5 wt.%, and a market-available GFRP bar with 9.5 mm diameter; (**b**) comparison of horizontal shear strength. The percentage shown above the error bar represents the improvement in horizontal shear strength of pultruded GFRP bars incorporating COOH-MWCNTs.

The shear load–displacement behavior of the neat pultruded GFRP bars and pultruded GFRP bars incorporating 0.5 wt.% COOH-MWCNTs is shown in Figure 11. It can be observed that, by incorporating 0.5 wt.% MWCNTs, the slope of the linear part of the horizontal shear load–displacement curves increased. A significant improvement in the horizontal shear strength by 111% was observed for pultruded GFRP incorporating 0.5 wt.% COOH-MWCNTs compared with neat GFRP bars pultruded using the same settings. This behavior can be attributed to the improvement in the load transfers between the fiber and matrix [63]. The five specimens tested for each pultruded GFRP type had less than a 2% coefficient of variation. The improvement in the horizontal shear strength of pultruded GFRP bars incorporating 0.5 wt.% COOH-MWCNTs compared with neat pultruded GFRP bars was proven to be statistically significant, with a 95% confidence level using the Student *t*-test. It is important to note that the reported shear strength of the pultruded GFRP bar seems lower than that reported by the manufacturers. The significant increase in shear strength of pultruded GFRP bars incorporating MWCNTs is based on the sole comparison with neat GFRP bars pultruded using the same settings. However, testing of a market-available GFRP bar using the same short beam test reported herein showed the market-available GFRP bar with a 9.5 mm diameter to have the same short beam shear behavior and similar shear strength to the GFRP pultruded bar, as shown in Figure 11.

**Figure 12:**
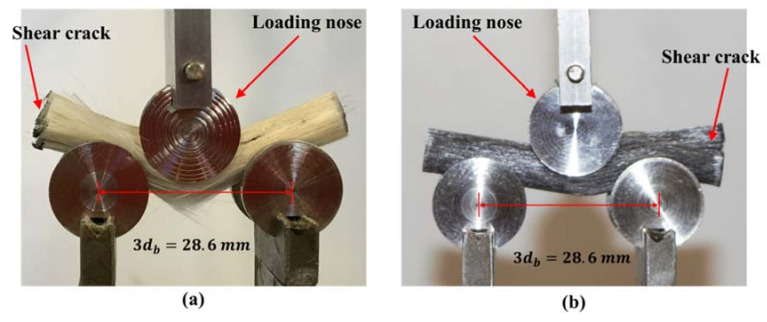
Short beam shear failure modes of pultruded GFRP specimens with (**a**) neat pultruded GFRP specimens and (**b**) pultruded GFRP specimens with 0.5 wt.% COOH-MWCNTs, showing shear cracks propagating in both specimens.

Failure modes of the short beam shear tests shown in Figure 12 indicate a brittle failure with interlaminar separations for the pultruded neat GFRP bar. Figure 12 shows shear cracks propagating in both specimens. Figure 12 also shows that GFRP bars incorporating 0.5 wt.% MWCNTs are laterally stiffer compared with neat GFRP bars. The increase in lateral stiffness of pultruded GFRP bars incorporating MWCNTs can be explained by the fact that the lateral stiffness of GFRP bars is governed by the matrix stiffness, which is affected by the inclusion of MWCNTs. The fiber snap and breakage at the midpoint of the specimen under the loading nose can be observed in Figure 12a. A nonbrittle interlaminar shear failure was observed for the pultruded GFRP bar incorporating 0.5 wt.% MWCNTs, as shown in Figure 12b. This behavior can be explained by the improvement in the bond strength and interlaminar fracture toughness of GFRP incorporating COOH-MWCNTs, as suggested above.

As the horizontal shear strength of the GFRP is a dominant matrix behavior, it is obvious that incorporating COOH-MWCNTs can significantly improve the horizontal shear strength of pultruded GFRP bars. The above results indicate that using as low as 0.5 wt.% COOH-MWCNTs well dispersed in the vinyl ester matrix prior to pultrusion of the GFRP bar could significantly improve the tensile strength by 20% and horizontal shear strength by 111% and change the GFRP modes of failure. A summary of the mechanical test results on pultruded neat GFRP bars and pultruded GFRP bars incorporating 0.5 wt.% COOH-MWCNTs is presented in Table 3. The significant improvement in horizontal shear strength of pultruded GFRP bars incorporating COOH-MWCNTs is specifically useful for producing GFRP reinforcing dowels in concrete slab on grade and in other applications where GFRP is typically not used for its limited shear strength.

**Figure 13:**
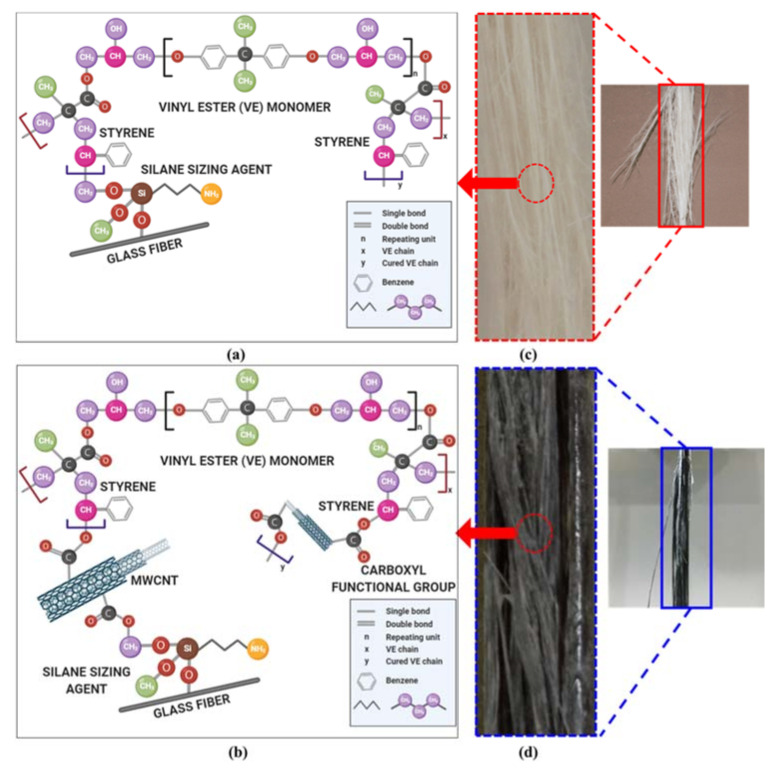
Chemical interactions in (**a**) neat pultruded GFRP bars [52,64,65], and (**b**) pultruded GFRP bars incorporating 0.5 wt.% COOH-MWCNTs. Relationship with the surface morphology of (**c**) broom failure in neat pultruded GFRP bars and (**d**) absence of broom failure in pultruded GFRP incorporating 0.5 wt.% COOH-MWCNTs.

The FTIR spectrographs confirmed the hypothesis that COOH-MWCNTs had chemical effects on the vinyl ester and, thus, resulted in an improved interaction with the glass fibers used in the pultrusion of GFRP reinforcing bars. The chemical structure of vinyl ester resin after initiation and cross-linking with a styrene monomer and its reaction with the silane sizing agent on the surface of glass fiber is shown schematically in Figure 13a [52,64,65]. The COOH functionalization group of the MWCNTs had a chemical reaction with the vinyl ester on one side and with the silane sizing of the glass fibers on the other side (Figure 13b). This interaction seemed to increase the bond strength between the vinyl ester matrix and the glass fibers, thus improving the horizontal shear strength of the pultruded GFRP reinforcing bars. A schematic representation of the chemical interaction of vinyl ester incorporating COOH-MWCNTs with the silane sizing agent on the glass fibers [26] used in pultruded GFRP reinforcing bars compared with neat vinyl ester interactions with the silane sizing agent in the pultruded GFRP reinforcing bars is shown in Figure 13.

Cost analysis of materials and equipment in the 2020 market shows that the cost to produce pultruded GFRP with vinyl ester incorporating 0.5 wt.% COOH-MWCNTs would be 10–15% higher than that of neat vinyl ester. The fact that the selected amount of MWCNTs does not affect viscosity or fiber wet-out means that no change is needed for the pultrusion process, with only a need to disperse the nanomaterials in the vinyl ester prior to pultrusion. An additional one-time investment will be required to incorporate the dispersion of MWCNTs in vinyl ester in the production line. Industrial-scale equipment for the dispersion of nanomaterials in polymer resins such as calendaring techniques is available and has been reported to work efficiently [66].

Further research is warranted to identify the absolute amount of MWCNTs to be used in a commercial application. MWCNTs contents below 0.5 wt.% (e.g., 0.4 or 0.2 wt.%) might still produce pultruded GFRP bars with the desired improvement in mechanical properties. Further research is also warranted to examine the fatigue, creep rupture, and durability behavior of pultruded GFRP incorporating MWCNTs. The fact that most of these properties are governed by the matrix and limitations of the bond of glass fibers with polymer resins suggests that the new pultruded GFRP bars incorporating COOH-MWCNTs might provide a good solution to these critical limitations in GFRP.

## 7. Conclusions

A pultrusion facility was developed to produce GFRP reinforcing bars with a novel nanomodified vinyl ester resin incorporating MWCNTs. Viscosity measurements and SEM investigations were first conducted to define the suitable content for MWCNTs dispersed in the vinyl ester resin that can be used in the pultrusion process. Investigations showed that contents above 0.5 wt.% suffer from poor dispersion or increased viscosity that would not allow fiber wet-out necessary for pultrusion. GFRP reinforcing bars were pultruded using neat vinyl ester resin and vinyl ester incorporating 0.5 wt.% COOH-MWCNTs. The COOH functionalization improved the dispersion of MWCNTs in the vinyl ester resin. Incorporating 0.5 wt.% functionalized COOH-MWCNTs in the vinyl ester prior to GFRP pultrusion improved the tensile strength of pultruded GFRP reinforcing bars by 20% and improved the horizontal shear strength of the bars by 111% compared with neat GFRP bars pultruded using the same settings. A significant change in the mode of failure of the GFRP bars was also observed, indicating an improved interlaminar bond of GFRP incorporating MWCNTs. The improved bond was attributed to the chemical reaction of COOH-MWCNTs with the vinyl ester and the resulting bond with the silane sizing on glass fibers. FTIR measurements confirmed the chemical reaction between COOH-MWCNTs and the vinyl ester. The quality of the pultruded GFRP reinforcing bars was confirmed by the high fiber volume fraction and high degree of cure. Cost analysis of the new pultruded GFRP reinforcing bars using nanomodified vinyl ester incorporating MWCNTs showed a limited cost increase considering the observed improvement in mechanical properties, specifically, the horizontal shear strength above 100% of neat GFRP bars. The improvements in the mechanical properties of GFRP allow widening the use of GFRP in applications requiring improved shear strength (e.g., reinforcing dowels for concrete slab on grade). Further research is warranted to examine the fatigue and creep rupture strength of the new pultruded GFRP reinforcing bars incorporating MWCNTs.

## 8. Patents

Mahmoud Reda Taha, Rahulreddy Chennareddy, Amr Riad Pultruded GFRP Reinforcing Bars, Dowels and Profiles with Carbon Nanotubes. PCT/US2018/059015. WO/2019/090120.

## Figures and Tables

**Table 1:** Viscosity measurements of the vinyl ester (VE) with varying MWCNTs contents.

Specimen	Viscosity (mPa·s)					Mean (mPa·s)	Standard Deviation (mPa·s)	Coefficient of Variation (%)
	1	2	3	4	5			
VE Neat (0.0 wt.%)	399.3	400.8	402.7	404.6	406.0	402.7	2.72	1.0
VE 0.5 wt.%	455.1	451.5	446.2	443.1	444.3	448.0	5.08	1.0
VE 1.0 wt.%	465.6	462.7	463.4	464.5	464.7	464.2	1.13	0.0
VE 2.0 wt.%	514.4	524.9	521.5	518.4	523.9	520.6	4.28	1.0

**Table 2:** Normalized heat of reaction and consequent degree of cure for uncured vinyl ester, neat cured vinyl ester, and cured vinyl ester incorporating 0.5 wt.% COOH-MWCNTs.

Specimen	Normalized Heat of Reaction (J/g)	Degree of Cure (%)
Uncured Vinyl Ester	577.56	-
Cured Vinyl Ester (Neat 0.0 wt.%)	13.10	97.73
Cured Vinyl Ester (0.5 wt.% COOH-MWCNTs)	10.98	98.10

**Table 3:** Elastic modulus, tensile strength, and horizontal shear strength of pultruded neat GFRP bars and pultruded GFRP bars incorporating 0.5 wt.% COOH-MWCNTs.

Specimen	Tensile Strength (MPa)	Tensile Modulus (GPa)	Horizontal Shear Strength (MPa)
Neat GFRP	694 ± 71	45.4 ± 0.29	24.6 ± 1.0
GFRP 0.5 wt.%	832 ± 42	45.5 ± 1.66	49.6 ± 2.4
